# First person – Chaitali Khan

**DOI:** 10.1242/dmm.052719

**Published:** 2025-12-29

## Abstract

First Person is a series of interviews with the first authors of a selection of papers published in Disease Models & Mechanisms, helping researchers promote themselves alongside their papers. Chaitali Khan is first author on ‘ Allograft transplantation for *Drosophila* tumor metastasis studies’, published in DMM. Chaitali is a postdoctoral fellow in the lab of Nasser M. Rusan at National Heart, Lung, and Blood Institute, Bethesda, MD, USA, investigating the fundamental molecular and cellular mechanisms governing the tumour–host interactions that facilitate cancer metastasis to distant organs.


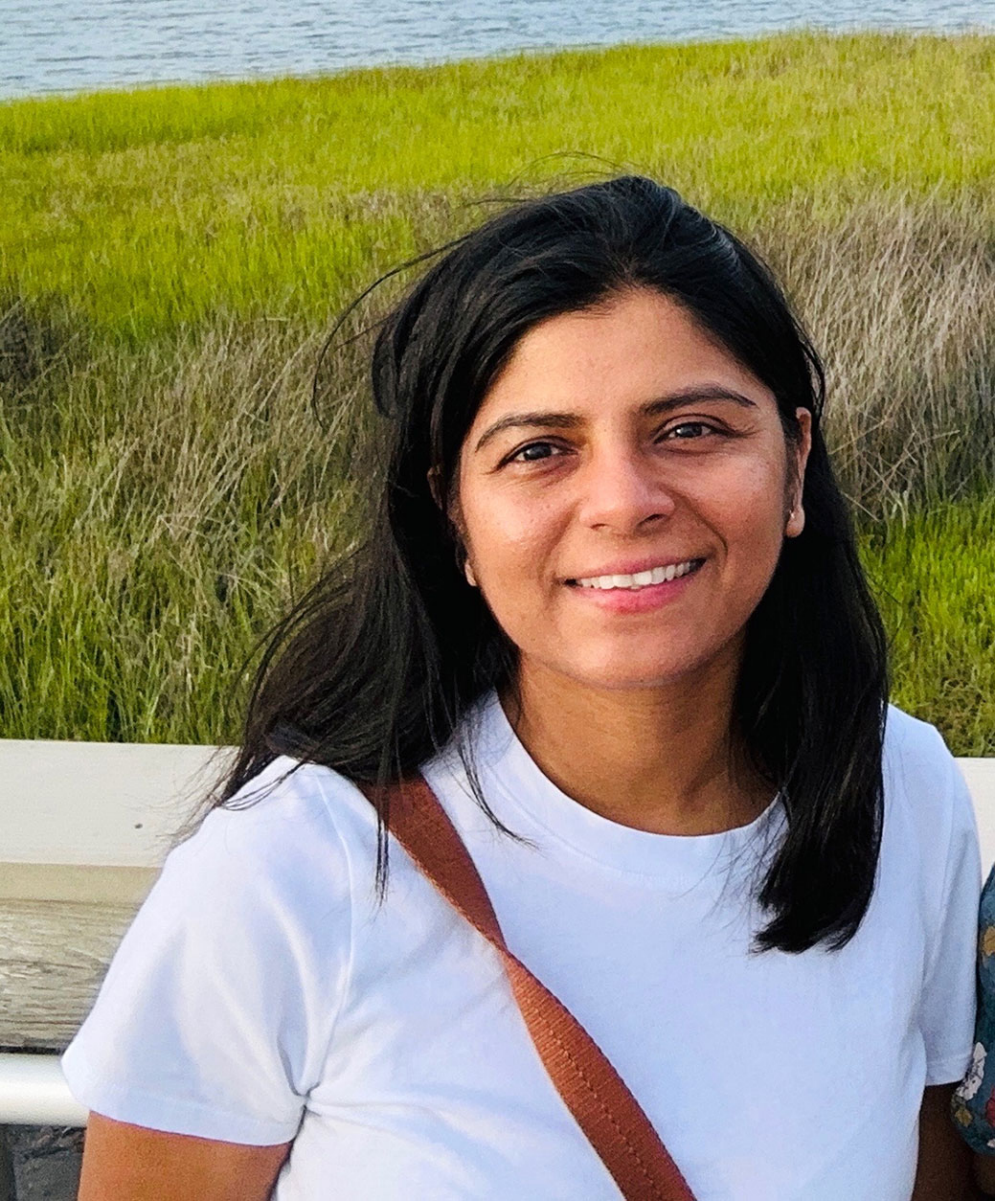


**Chaitali Khan**

**Who or what inspired you to become a scientist?**

My early childhood was shaped by the joy of experimental and exploratory science, fostered by backyard experiments with my elder brother. My genuine interest in genetics was later sparked by Professor S. Lakshmi Devi, head of the college, who taught with infectious passion. Walking through the Delhi University campus and seeing the sweet pea plants, which I believe were planted at her request, I realized the mathematical logic inherent in genetic experiments. This appreciation led me to choose the *Drosophila* model throughout my research career, which I consider to be unequivocally the best system for genetic studies.

**What is the main question or challenge in disease biology you are addressing in this paper? How did you go about investigating your question or challenge?**

This paper's core objective was to develop a high-throughput method for generating and studying metastasis in distant organs of adult *Drosophila*, thereby enabling us to address fundamental questions in cancer biology, such as cancer invasion and colonization. This work builds upon the pioneering allograft transplantation studies by Elizabeth Gateff, which decades ago led to the isolation of the first tumour suppressor gene, *lethal giant larvae* (*lgl*). While allograft transplantation has been used extensively to characterize the tumorigenicity of various genetic mutants, less work has been done using it for large-scale metastasis studies. So, it was basically an uncharted area when I started this project, and I had to play around to understand what to expect and how to establish it as a workable platform. It was both fun and challenging to successfully establish and precisely define transplantation stages at a level that permits rapid, large-scale studies.

**How would you explain the main findings of your paper to non-scientific family and friends?**

This paper talks about cancer metastasis, the process that allows a cancer cell (like one from ovarian cancer) to break away and thrive in a completely new organ, such as the brain. This process involves cancer cells entering the brain tissue and multiplying and forming cancer colonies, which is often referred to as Stage IV cancer, and could be a deadly outcome for cancer patients. Because we can't easily study this in humans, I use fruit flies as a simple and powerful model system. This allows me to create and observe cancer cells traveling in real time, and simultaneously enables studies of the organ to which cancer has spread. I use this information to design genetic experiments that will help find genes that are crucial in driving this process.

… this work is designed to identify novel therapeutic targets that can disrupt the metastatic cascade, driving forward translational research for the benefit of human patients

**What are the potential implications of these results for disease biology and the possible impact on patients?**

In this work, I successfully established a powerful platform that allows the real-time study of metastasis in *Drosophila* organs, such as the brain and ovaries. These innovative models offer two crucial advantages: they enable us to observe the dynamic cellular interplay between cancer and host tissues, providing vital insights into how cancer cells breach natural tissue barriers. Furthermore, they are essential for defining the specific migratory and invasive mechanisms utilized by metastatic cells. Combined, these models offer an unparalleled, fast-paced system for uncovering fundamental cellular and molecular mechanisms that are conserved across species. Ultimately, this work is designed to identify novel therapeutic targets that can disrupt the metastatic cascade, driving forward translational research for the benefit of human patients.

**Figure:**
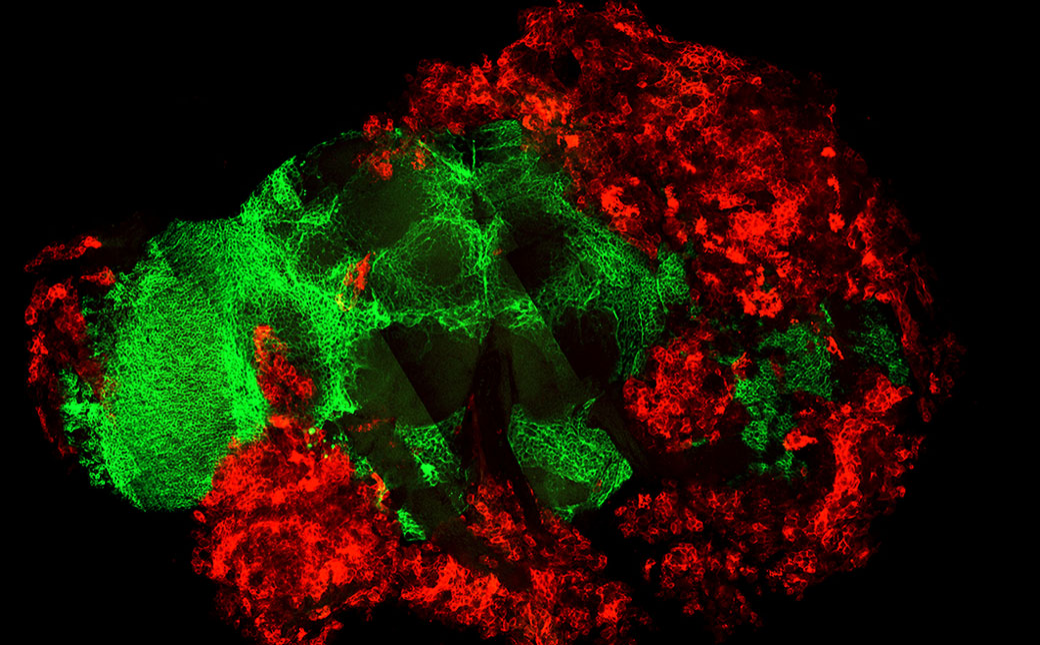
Confocal image of metastasized cancer cells (red) within the adult *Drosophila* brain (green).

**Why did you choose DMM for your paper?**

Given that the central theme of this paper was modelling cancer metastasis in the *Drosophila* model system, we couldn't think of a better fit than Disease Models & Mechanisms (DMM). DMM's visibility made it the best place to showcase our work. In fact, my prior positive experience publishing in the Journal of Cell Science, where I was particularly impressed by the excellent peer review and the post-acceptance services, further solidified my commitment to publishing future research with The Company of Biologists’ journals.

**Given your current role, what challenges do you face and what changes could improve the professional lives of other scientists in this role?**

The major challenge I face as a *Drosophila* biologist is the persistent need to justify the fruit fly model, despite its history of foundational contributions. Decades of research have established *Drosophila*’s critical role in discovering genes and signalling pathways central to human cancer biology. I believe *Drosophila*’s excellent genetics and a short life cycle make it perfect for making fundamental discoveries at a fraction of the time and cost of other models. In today's era of massive, high-throughput data generation, the fly's role is not to replace human studies, but to inform them. The fundamental findings generated by *Drosophila* are perfectly positioned to drive informative studies, turning vast datasets into actionable biological hypotheses that can fast track the translational research and contribute to clinical breakthroughs.

… the fly's role is not to replace human studies, but to inform them

**What's next for you?**

I'm currently finalizing a project on cancer invasion, leveraging the *Drosophila* brain metastasis model that I established in this paper. The broad applicability of this novel platform to dissect the fundamental mechanisms of metastasis has positioned me to start my own independent research program. I am now seeking faculty positions to begin another exciting journey.

**Tell us something interesting about yourself that wouldn't be on your CV**

Besides my research, I enjoy listening to audiobooks across a range of topics from non-fiction to classic literature. I love to cook in the evenings as a way to unwind, and I am inspired by how deep flavours can be created with just a few ingredients. This passion has recently turned into a collaborative effort with my mother to compile our family's authentic day-to-day Mughlai cuisine. This is not the elaborate, royal banquet fare often publicized, but the simple, authentic cooking we enjoy daily with simple ingredients.
